# PDCD4 as a marker of mTOR pathway activation and therapeutic target in mycobacterial infections

**DOI:** 10.1128/spectrum.00062-24

**Published:** 2024-06-24

**Authors:** Ruchi Paroha, Jia Wang, Sunhee Lee

**Affiliations:** 1 Department of Microbiology and Immunology, The University of Texas Medical Branch, Galveston, Texas, USA; University of Nebraska Medical Center, Omaha, Nebraska, USA

**Keywords:** mycobacteria, PDCD4, mTOR, FDA-approved drug library and drug screening

## Abstract

Programmed cell death protein 4 (PDCD4) is instrumental in regulating a range of cellular processes such as translation, apoptosis, signal transduction, and inflammatory responses. There is a notable inverse correlation between PDCD4 and the mammalian target of rapamycin (mTOR) pathway, which is integral to cellular growth control. Activation of mTOR is associated with the degradation of PDCD4. Although the role of PDCD4 is well established in oncogenesis and immune response regulation, its function in mycobacterial infections and its interplay with the mTOR pathway necessitate further elucidation. This study investigates the modulation of PDCD4 expression in the context of mycobacterial infections, revealing a consistent pattern of downregulation across diverse mycobacterial species. This observation underscores the potential utility of PDCD4 as a biomarker for assessing mTOR pathway activation during such infections. Building on this finding, we employed a novel approach using PDCD4-based mTOR (Tor)-signal-indicator (TOSI) reporter cells for the high-throughput screening of FDA-approved drugs, focusing on mTOR inhibitors. This methodology facilitated the identification of several agents, inclusive of known mTOR inhibitors, which upregulated PDCD4 expression and concurrently exhibited efficacy in impeding mycobacterial proliferation within macrophages. These results not only reinforce the significance of PDCD4 as a pivotal marker in the understanding of infectious diseases, particularly mycobacterial infections, but also illuminate its potential in the identification of mTOR inhibitors, thereby contributing to the advancement of therapeutic strategies.

**IMPORTANCE:**

This study emphasizes the critical role of the mammalian target of rapamycin (mTOR) pathway in macrophage responses to mycobacterial infections, elucidating how mycobacteria activate mTOR, resulting in PDCD4 degradation. The utilization of the (Tor)-signal-indicator (TOSI) vector for real-time monitoring of mTOR activity represents a significant advancement in understanding mTOR regulation during mycobacterial infection. These findings deepen our comprehension of mycobacteria's innate immune mechanisms and introduce PDCD4 as a novel marker for mTOR activity in infectious diseases. Importantly, this research laid the groundwork for high-throughput screening of mTOR inhibitors using FDA-approved drugs, offering the potential for repurposing treatments against mycobacterial infections. The identification of drugs that inhibit mTOR activation opens new avenues for host-directed therapies, marking a significant step forward in combating tuberculosis and other mycobacterial diseases.

## INTRODUCTION

Tuberculosis (TB), a historic disease caused by the pathogen, *Mycobacterium tuberculosis*, was responsible for a staggering 1.6 million deaths, making it the second most prevalent infectious disease globally, following only COVID-19, according to the Global Tuberculosis Report 2021. Such statistics underscore a critical demand for innovative therapeutic solutions to address the rising challenges of TB. These challenges encompass the emergence of drug-resistant strains, the need to enhance treatment outcomes, and the urgency to shorten therapeutic durations. Numerous strategies are currently under investigation to address this critical need, including new drug discovery, combination therapies, host-directed therapies, and the repurposing of existing drugs (1–3).

A hallmark of *M. tuberculosis* is its skill in evading host immune responses. This microbe possesses a repertoire of mechanisms that allow it to persist within macrophages without elimination (4). Such strategies range from hindering phagosomal maturation to manipulating host signaling pathways, including those related to cell death like autophagy and apoptosis (5, 6). Multiple virulence factors of *M. tuberculosis* serve to impede autophagy primarily by disrupting the fusion of phagosomes with lysosomes, a critical step in the autophagic process. Notably, proteins such as enhanced intracellular survival (Eis), early secreted antigenic target 6 (ESAT-6), protein tyrosine phosphatase A (PtpA), and protein kinase G (PKnG) are secreted by *M. tuberculosis* into phagosomes, where they exert inhibitory effects on autophagosome formation and maturation (7, 8). By subverting host autophagy, *M. tuberculosis* enhances its intracellular survival and persistence, facilitating the establishment and progression of tuberculosis infection (9). Mycobacteria also regulate apoptosis through a combination of strategies that involve manipulation of host signaling pathways, modulation of death receptors and anti-apoptotic proteins, induction of immune responses, and activation of intracellular survival mechanisms (10–12). Understanding these complex host–pathogen interactions at a molecular level is critical for innovating therapeutics, encompassing novel drug targets, immunomodulatory interventions, and precision medicine strategies (13).

Central to many cellular processes, the mammalian target of rapamycin (mTOR) and its complexes, mTORC1 and mTORC2, govern cellular metabolism, protein synthesis, cell death, and autophagy (14). The mTOR pathway, influenced by diverse triggers such as immune signals and environmental stress, integrates with networks like PI3K/AKT and TSC1/TSC2 to regulate cellular functionalities, including protein synthesis and catabolism (15, 16). Intriguingly, the mTOR-autophagy axis plays a central role in dictating the outcomes of host defense against mycobacterial infections (17–19).

Monitoring mTOR’s activity, especially in autophagy, is essential to developing therapies that amplify immune responses. Evidence suggests that mycobacteria exploit disruptions in autophagy to facilitate their survival and replication, thus making the process of pathogen clearance more complex (20, 21). Our group has documented mTOR pathway activation during mycobacterial infections (22, 23). With this in mind, strategies targeting mTOR signaling or autophagy emerge as potential counters to mycobacterial infections (24, 25). One promising avenue is the repurposing of drugs to activate autophagy as a therapeutic approach against *M. tuberculosis* (20).

To better understand mTOR activity during these infections, we used the mTORC (Tor)-signal-indicator (TOSI), a tool developed by Oki et al., which utilizes PDCD4, a downstream target of mTOR (26, 27). Although initially crafted for acute myeloid leukemia, our findings suggest TOSI’s potential for studying mTOR signaling in mycobacterial infection settings. PDCD4, an integral protein involved in cell survival and widely acknowledged as a tumor suppressor, has diverse roles in immune and non-immune cells (28, 29). Its expression is influenced by various factors and has been associated with cancer progression, metabolic disorders, and inflammatory responses (30–33). Recognizing its potential role in regulating intracellular pathogen survival (34), we sought to probe the interplay of PDCD4 and the mTOR pathway during mycobacterial infections.

In the current study, our primary objective revolves around elucidating the modulation of the mTOR/PDCD4 axis within mycobacterial-infected macrophages. Employing TOSI-based reporter cell lines, we have optimized these cells for a high-throughput assessment of an FDA-approved drug library, aiming for a precise evaluation of potential drug candidates and their impacts on mTOR regulation and autophagy. Our rigorous efforts have identified a few novel mTOR inhibitors as promising host-directed therapeutic candidates for TB.

## MATERIALS AND METHODS

### TOSI reporter cell lines

**Fig 1:**
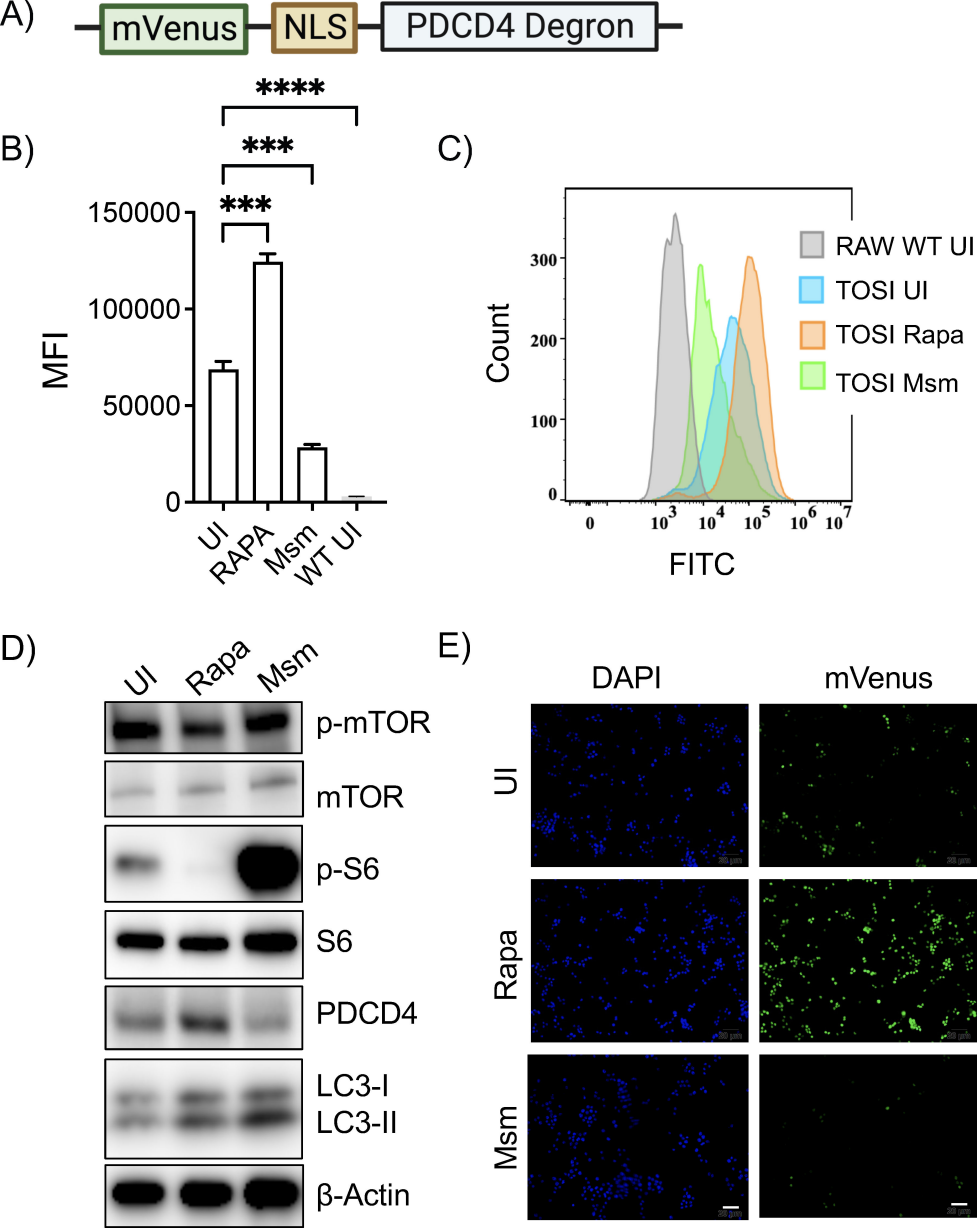
Mycobacterial infection induces degradation of PDCD4 in macrophages. (**A**) The TOSI vector is depicted schematically, highlighting the incorporation of a PDCD4 degron equipped with an NLS and mVenus to monitor PDCD4 expression. (**B**) Following a 3-hour infection with *M. smegmatis* at an MOI of 10, TOSI cells were analyzed using flow cytometry. For comparative purposes, a separate cohort of cells was treated with 5 µM rapamycin. For each sample, a minimum of 10,000 cells were acquired and analyzed. Uninfected RAW 264.7 WT cells are included as a control. (**C**) The histogram in this panel illustrates the data obtained from panel B. RAW 264.7 WT cells are used as a baseline for fluorescence, which is depicted in gray. (**D**) We explored the dynamics of intrinsic PDCD4 degradation upon mTOR activation by performing Western blot analyses on cell lysates post-infection. The analysis covered the expression of PDCD4, mTOR, p-mTOR, pS6, S6, and LC3-I/II. Rapamycin was utilized as a control, and β-actin served as the loading control. (**E**) Fluorescence microscopy was employed to display the levels of PDCD4 expression after infection or following 5 µM rapamycin treatment. DAPI staining was used for nucleus visualization, while mVenus intensity was indicative of PDCD4 expression levels. These data are representative of three separate experiments. Scale bars = 20 µm. Statistical evaluations were performed using a one-way ANOVA, followed by Dunnett’s test for multiple comparisons, where ***P ≤ 0.001 and ****P ≤ 0.0001. UI, uninfected; Msm*, M. smegmatis*; FITC, fluorescein isothiocyanate.

Raw 264.7 cells, used in creating our TOSI reporter lines, were cultured in Dulbecco's modified Eagle's medium (DMEM) media supplemented with 10% fetal bovine serum (FBS), non-essential amino acids, 10 mM HEPES, and 50 µM β-mercaptoethanol. The RAW 264.7 cells are often used along with different macrophage models such as THP-1, BMDM, J774A.1 cell lines to study mycobacterial infection. The culture conditions were maintained at 37°C in an atmosphere of 5% CO_2_. The TOSI construct, designated pMXs-IP-mVenus-TOSI, was graciously provided by David Scadden (Addgene plasmid #172491). This vector encompasses mVenus linked to the N-terminal 80 amino acids of the PDCD4 degron (Fig. 1A). Using this reporter, the inverse relationship between mVenus fluorescence intensity and mTORC1 activity was quantified via flow cytometry and microscopy.

The TOSI vector was transfected into the Raw 264.7 cells when they reached a confluency of 70%–90%, utilizing the Lipofectamine 2000 Transfection Reagent as per the manufacturer’s recommendations. Following the transfection, cells were incubated for an additional 24 hours prior to media exchange with fresh DMEM. After a subsequent 48-hour period, the transfected cells underwent selection by adding 10 µg/mL puromycin to the growth medium. This selection process facilitated the enrichment of cells that had successfully integrated the TOSI vector. Once selected, the puromycin-resistant cells were expanded and subsequently sorted into individual wells of a 96-well plate via fluorescence-activated cell sorting (FACS) for clonal expansion. Ensuring maintenance of the TOSI integration, these clonally expanded cells were continually cultured under antibiotic selection.

To ascertain the effectiveness of the newly developed cell lines, we subjected them to two specific experimental conditions: infection with *Mycobacterium smegmatis* mc^2^155, which is an activator of the mTOR pathway, and administration of rapamycin, a recognized mTOR pathway inhibitor. This dual approach successfully demonstrated the cell lines’ capability to respond to distinct mTOR pathway modulations, thereby validating their applicability for future research endeavors.

### Mycobacterial infection in macrophages

*M. smegmatis* mc^2^155 was cultured in 7H9 media, enriched with 0.5% glycerol and 0.02% tyloxapol. Similarly, BCG Danish, *M. tuberculosis* H37Ra, *M. tuberculosis* H37Rv, and Erdman strains were propagated in the same media, but with an added 10% oleic albumin dextrose catalase (OADC) supplement. Virulent mycobacterial strains such as *M. tuberculosis* H37Rv and Erdman were handled within a biosafety level 3 (BSL3) laboratory. These cultures were incubated at 37°C until they achieved an optical density at 600 nm (OD_600_) between 0.6 and 0.8. Mycobacterial cultures were centrifuged at 2,000 × *g* for 10 minutes, washed with 1× phosphate-buffered saline (PBS), and resuspended in DMEM media. The mixture was centrifuged at 800 × *g* for 8 minutes to ensure a homogenous bacterial suspension. The bacterial concentration for infection was gauged by assessing the OD_600_. Macrophages were plated in 12-well plates at a density of 2.5 × 10^5^ cells/mL and were allowed to adhere for 24 hours under conditions of 37°C and 5% CO_2_. The prepared single-cell bacterial suspension was then introduced to the macrophages at a multiplicity of infection (MOI) of 10 unless specified otherwise and was allowed to interact for up to 3 hours. This was followed by triple washing with PBS to remove unbound bacteria. Subsequently, a solution of 50 µg/mL gentamicin in DMEM was added to the macrophages for 1 hour to eradicate any remaining extracellular bacteria. After this treatment, macrophages were washed thrice with PBS and were maintained in DMEM supplemented with 20 µg/mL gentamicin for designated intervals.

### CFU assays

TOSI cells were infected with *M. tuberculosis* H37Ra at an MOI of 10 to evaluate the efficacy of the selected drug candidates. Following infection, the cells underwent treatment with the selected drug compounds for a duration of 24 hours. After this, the cells were washed with PBS and lysed with radioimmunoprecipitation assay (RIPA) buffer to harvest the intracellular bacteria. The resulting cell lysate was serially diluted and plated onto 7H10 agar plates enriched with OADC. These plates were subsequently incubated at 37°C for 4 weeks.

### Live-dead staining

TOSI cells were either uninfected/untreated, or infected with *M. smegmatis* at an MOI of 5 for 3 hours, or treated with rapamycin at 1 µg/mL for overnight. The next day, the cells were harvested and stained with FVD660, and then fixed. The cells were acquired on BD symphony.

### Flow cytometry

After the infection and/or treatment, cells were washed with PBS and were detached using 0.25% trypsin-EDTA (Gibco). Subsequently, cells underwent another PBS wash and were fixed using 4% paraformaldehyde. Post-fixation, cells were washed once more with PBS. Samples were then analyzed using a BD Accuri C6 Plus flow cytometer to assess mVenus expression and TOSI responsiveness across varied experimental settings.

### Microscopy

For microscopic observation, cells were seeded onto coverslips and then infected with the mycobacterial strains. Post-infection, cells were briefly washed with PBS and were fixed using 4% paraformaldehyde for 15 minutes. Cells were then washed again with PBS and were affixed onto slides using ProLong Gold Antifade reagent containing DAPI (4′,6-diamidino-2-phenylindole; Cell Signaling Technologies). Slides were subsequently visualized under an Olympus microscope, and image acquisition was facilitated by the Cellsense software.

### Immunoblotting

Cellular proteins were extracted by lysing the cells with RIPA buffer, supplied by Sigma-Aldrich. Following lysis, the mixture was centrifuged at 15,000 rpm for 15 minutes to clarify the lysate. The resultant supernatant was mixed with 4X Laemmli buffer and then heated at 95°C for 5 minutes to denature the proteins. A 12% SDS-PAGE gel was prepared to facilitate protein separation. Denatured proteins were subsequently loaded onto this gel. After electrophoresis, proteins were transferred onto polyvinylidene fluoride (PVDF) membranes procured from Millipore (Burlington, MA, USA). Post-transfer, these membranes were blocked for 1 hour to prevent non-specific protein binding. Primary antibodies, including anti-LC3B (catalog no. 2775), anti-phospho-S6 (Ser235/236; no. 4857), anti-S6 (no. 2217), and anti-β-actin (no. 4970), all sourced from Cell Signaling Technology (Danvers, MA, USA), were incubated with the membranes. For detection, an anti-rabbit IgG horseradish peroxidase (HRP)-linked secondary antibody (Cell Signaling Technologies, catalog no. 7074) was used. Protein bands were revealed using the Clarity Western ECL Substrate from Bio-Rad. Band images were captured with an Amersham Imager 680 from GE Healthcare Life Sciences (Marlborough, MA, USA). Subsequent image analysis was performed using the ImageJ software from the National Institutes of Health (NIH).

### Screening of FDA-approved drug library

*M. tuberculosis* H37Ra-tdTomato was cultured in 7H9 medium supplemented with OADC and 50 µg/mL hygromycin until reaching an optical density (OD600) between 0.6 and 0.8. Following this, TOSI cells were infected with this mycobacterial suspension at an MOI of 10. The infected culture was incubated for 2 hours at 37°C in a 5% CO_2_ atmosphere with shaking at 130 rpm. Post-incubation, cells were centrifuged and treated with DMEM complete medium containing 50 µg/mL amikacin for 1 hour with shaking to eliminate residual extracellular bacteria. After three washes with antibiotic-free media, cells were resuspended in DMEM complete medium and were plated at a density of 1 × 10^5^ cells/well in 96-well black assay plates with transparent bottoms (Corning). Before cell addition, each well was loaded with 110 µM of various drugs from the Enzo SCREEN-WELL FDA-approved drug library V2, achieving a final concentration of 10 µM post-cell addition.

At 24 hours post-infection, media were carefully removed, and cells were fixed with 4% paraformaldehyde (PFA) at room temperature for 15 minutes. After a PBS wash, cells were stained with Hoechst (1:2,000 dilution) for 5 minutes at room temperature. Following another PBS wash, cells were prepared for imaging in PBS. Imaging was performed on five fields per well using the 10X objective in confocal mode on the Opera High-Content Imaging System (PerkinElmer). Data were processed using Harmony 5.0 software to quantify mVenus expression in TOSI cells, tdTomato for mycobacterial infection, and Hoechst for nuclear staining.

Post two independent screenings, a list of the top 50 drug candidates that demonstrated the highest fluorescent levels (indicative of mTOR inhibition) compared to untreated/infected controls was compiled. Recurrent drugs in both screenings were subjected to further validation in a secondary screen. Their efficacy was validated through Western blot analysis. The Z'-factor for assay quality assessment was calculated using the formula: Z'-factor = 1 – [(3σ_c+ + 3σ_c–)/|μ_c+ – μ_c–|], where σ represents the standard deviation, and μ denotes the mean value of the positive (c+) and negative (c–) controls.

### Statistical analysis

Data are expressed as mean ± standard deviation (SD), calculated from two or three independent experiments. Statistical evaluations were performed using a one-way analysis of variance (ANOVA), supplemented with Dunnett’s multiple-comparison test for individual comparisons. For grouped data analyses, a two-way ANOVA was applied, followed by Dunnett’s multiple-comparison test for post-hoc analysis. Statistical significance was established at a *P* value of ≤0.05. All statistical analyses were conducted using GraphPad Prism software.

## RESULTS

### Activation of mTOR by mycobacteria leads to PDCD4 degradation in macrophages

We previously elucidated that the mTOR pathway is activated following mycobacterial infection, initiating a series of events, notably the activation of pS6. As depicted in Figure S1, this pS6 activation leads to the phosphorylation of PDCD4, which subsequently recruits ubiquitin (Ub) via the βTRCP ubiquitin ligase. Once tagged by Ub, PDCD4 is targeted to the proteasome complex for degradation (35). Prior research has indicated a decline in PDCD4 levels during *Mycobacterium avium* infection, a process governed by miRNA 150 in RAW macrophages (36). Nonetheless, the function of PDCD4 as a reflection of mTOR activity during mycobacterial infections had not been explored.

To probe this aspect, we employed the TOSI vector (Fig. 1A), a construct with a PDCD4 degron fused to a nucleus localization sequence (NLS) and succeeded by mVenus. This allowed us to monitor mTOR activity during mycobacterial infections. RAW 264.7 cells, once transfected with the TOSI vector, were subjected to various stimuli to measure mTOR activity. In cells infected with *M. smegmatis*, a known potent mTOR activator, for 3 hours, we noted a marked decrease in PDCD4 levels compared to uninfected controls. Notably, upon the addition of rapamycin, a potent mTOR inhibitor, we observed increased PDCD4 levels (Fig. 1B and C). Treatment with rapamycin not only enhanced the native PDCD4 levels but also increased the levels of mVenus-fused PDCD4, as indicated by the mVenus mean fluorescence intensity (MFI) measurements. Our live–dead staining with FVD660 additionally verified that the increased mVenus response is not due to cell debris or treatment/infection-induced cell death but rather from mTOR activation (Fig. S2).

Further verification came from Western blot data, highlighting a surge in pS6 expression upon mTOR activation by mycobacteria, leading to diminished PDCD4 levels (Fig. 1D). Expectedly, rapamycin application caused a notable decrease in p-mTOR and diminished p-S6 levels, while S6 kinase remained unchanged. Elevated LC3BII levels further hint at the induction of the autophagy pathway. The decline in PDCD4 levels robustly indicates mTOR pathway activation in response to mycobacterial infections, further validated by the heightened pS6 levels in infected cells. Moreover, our findings concur with previous studies, suggesting that mycobacteria can concurrently activate both the mTOR and autophagy pathways, implying an mTOR-independent canonical autophagy activation during such infections.

Supplementing our findings, microscopic examinations revealed a distinct reduction in PDCD4 protein levels in *M. smegmatis-*infected TOSI cells compared to uninfected controls (Fig. 1E). As expected, rapamycin treatment increased PDCD4 levels in these cells. We observed a progressive degradation of PDCD4 in TOSI cells following exposure to *M. smegmatis*, with this degradation becoming more pronounced over time (Fig. S3). The correlation between PDCD4 and pS6 levels underscores the potential of PDCD4 as an alternative marker for mTOR activity in macrophages during mycobacterial infections. Our insights also pave the way to explore PDCD4’s role beyond oncological contexts, potentially emphasizing its pertinence in mycobacterial-induced infectious diseases.

### Species- and time-dependent degradation of PDCD4 induced by mycobacteria

**Fig 2:**
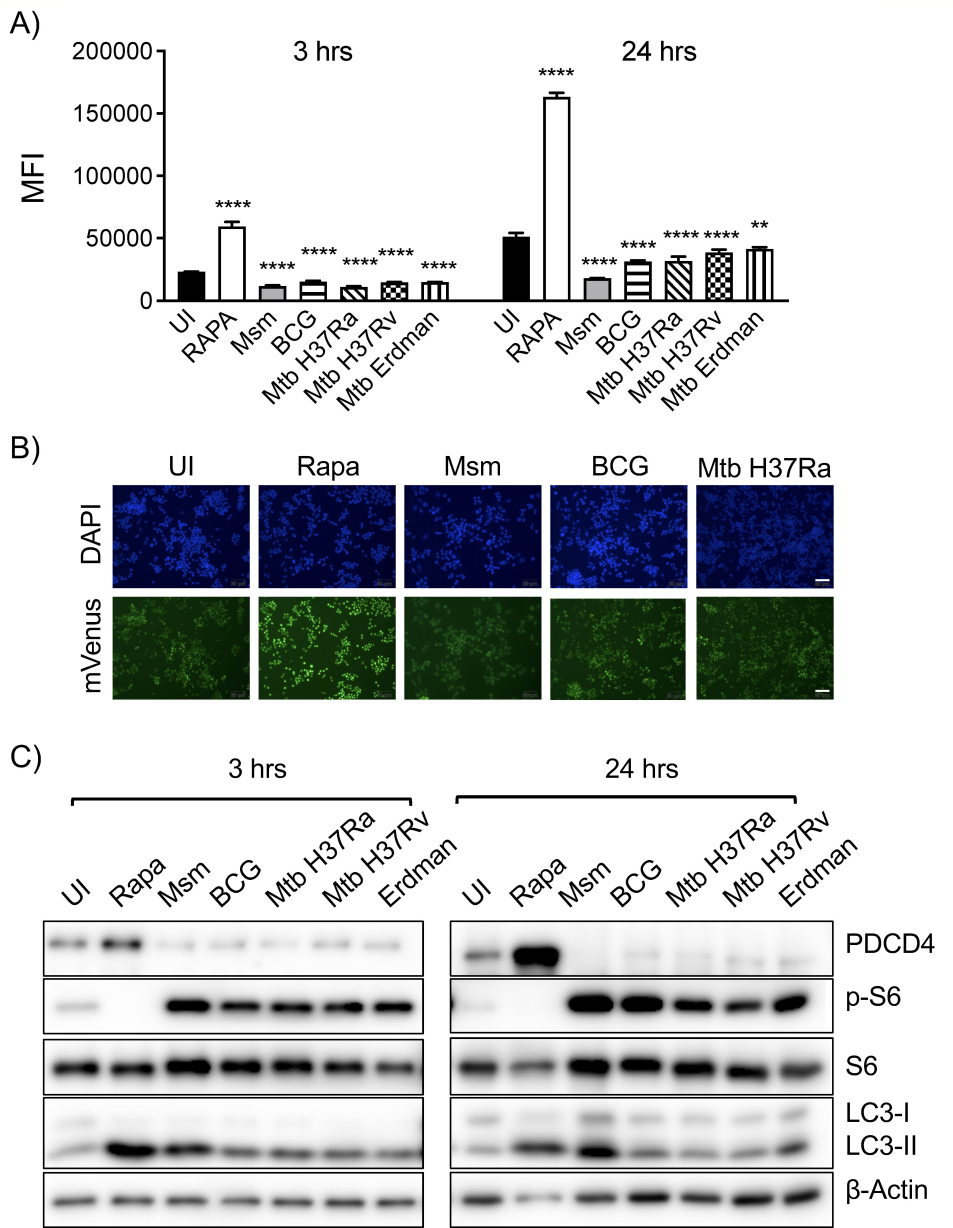
Examination of PDCD4 expression during mycobacterial infection. (**A**) PDCD4 expression in TOSI cells, indicated by mVenus MFI, was assessed at 3- and 24-hours post-infection with *M. smegmatis*, BCG, *M. tuberculosis* H37Ra, *M. tuberculosis* H37Rv, and Erdman strains using flow cytometry. (**B**) Microscopic analyses were conducted on TOSI cells 24 hours after infection, which also included treatments with 5 µM rapamycin. DAPI and mVenus fluorescence were utilized for visualizing nuclei and PDCD4, respectively. Scale bars = 20 µm. (**C**) Post-infection, Western blot analyses were performed to quantify proteins such as pS6, PDCD4, and LC3 in TOSI cell lysates at both time points post-infection. Rapamycin served as a control, and β-actin was used as a loading benchmark. Statistical analysis was conducted using one-way ANOVA with Dunnett’s multiple comparisons test, indicating significance levels of **P ≤ 0.01 and ****P ≤ 0.0001.

To determine whether PDCD4 degradation is a common response to different mycobacterial species, we expanded our investigation to include slow-growing strains such as *Mycobacterium bovis* (BCG), *M. tuberculosis* H37Ra along with virulent and highly pathogenic strains such as *M. tuberculosis* H37Rv and Erdman strains. Our findings revealed a uniform trend in PDCD4 expression levels in TOSI cells infected with these strains at both 3 and 24 hours post-infection, highlighting the widespread and pronounced activation of mTOR activity and subsequent PDCD4 degradation in various mycobacterial infections (Fig. 2A). Notably, the degradation of PDCD4 was more evident at the 24-hour mark with slow-growing mycobacteria, as confirmed by flow cytometry and microscopy (Fig. 2A and B). This could be a consequence of their inherently slower growth rates, possibly leading to a delayed response. To ensure consistency in the experiments, infection levels were standardized across different bacterial strains by quantifying the colony-forming units (CFU) of each bacterial culture used for inoculation. Comparative analysis revealed that *M. tuberculosis* H37Ra showed less PDCD4 degradation than *M. smegmatis* at 24 and 48 hours (Fig. S4).

To corroborate the endogenous levels of PDCD4, we compared our flow cytometry and microscopy data with Western blot results from RAW cells infected with various mycobacteria after 3 and 24 hours of infection (Fig. 2C). Densitometry data clearly indicated that post-infection, there was a notable decrease in PDCD4 expression coupled with an increase in phosphorylated S6 (pS6) levels, indicative of mTOR pathway activation (Fig. S5). The strong correlation between PDCD4 and pS6 expression, along with consistent results across different mycobacterial species, suggests that PDCD4 is a reliable alternative marker for assessing mTOR activity during mycobacterial infections.

### Assay validation for measuring mTOR activity using TOSI reporter cell lines with known mTOR modulators

In our pursuit of precise PDCD4 level measurements during mycobacterial infection, we conducted experiments with 20 replicates each of positive controls (rapamycin treated) and negative controls (untreated) to evaluate the TOSI cell line’s suitability for high-throughput screening (HTS). This assessment aimed to determine the TOSI cells’ ability to differentiate signals between these control groups effectively. We determined a Z′-factor of 0.74 based on the mean and standard deviation values from these experiments (Fig. S6). The Z′-factor, a widely accepted parameter, quantifies the extent of signal separation between positive and negative controls while considering the associated errors for each control.

**Fig 3:**
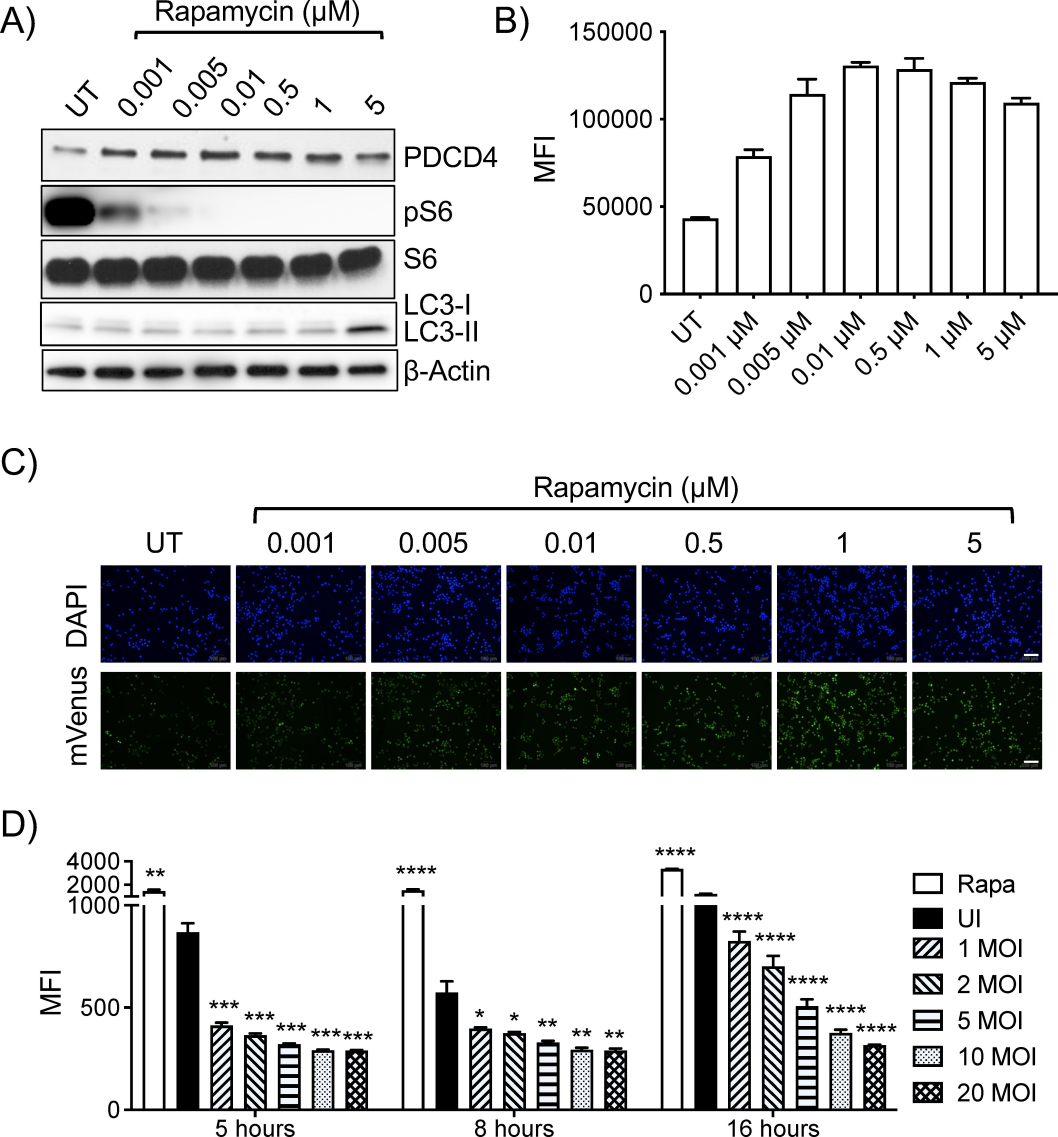
Dose-dependent responses in TOSI cells to mTOR inhibitors and activators. (**A**) Western blot analysis shows pS6/S6, PDCD4, and LC3 levels in TOSI cell lysates treated with different rapamycin concentrations for 3 hours; β-actin was the loading control. (**B**) Flow cytometry assessed mVenus MFI in TOSI cells after 3-hour rapamycin treatments at varying concentrations. (**C**) Microscopic images depict PDCD4 expression, indicated by mVenus intensity, in TOSI cells treated with varied rapamycin doses. Scale bars = 100 µm. (**D**) Following infections at different MOIs with *M. smegmatis*, mVenus levels in TOSI cells were recorded using the Opera high-content imaging system at designated intervals. The data presented here are representative of findings from a minimum of two separate experiments. For statistical analysis, a two-way ANOVA was conducted, followed by Dunnett’s test for post-hoc comparisons. The levels of statistical significance are denoted as follows: *P ≤ 0.05, **P ≤ 0.01, ***P ≤ 0.001, ****P ≤ 0.0001.

To further validate our approach, we generated dose–response curves for a well-known mTOR inhibitor, rapamycin. These findings were validated through Western blot analysis, which illustrated a concurrent dose-dependent inhibition of pS-6 and subsequent stabilization of PDCD4 (Fig. 3A). Despite the reduction in pS6 levels, the total S6 remains unchanged. Notably, the concentration-dependent increase in LC3-II expression, which is reflecting autophagy induction particularly evident at 5 µM, affirmed the suitability of the TOSI cell line for our HTS. This heightened sensitivity extends to the detection of minor changes in mTOR activity, a task that may prove challenging with traditional Western blotting methods. Flow cytometry data and microscopic analysis further demonstrated a dose-dependent increase in PDCD4 levels in the TOSI cells following treatment with varying concentrations of rapamycin (Fig. 3B and C).

In a parallel assessment, we validated our findings using *M. smegmatis*, a recognized mTOR activator (22, 23). TOSI cells underwent exposure to *M. smegmatis* infection at various MOIs and for differing periods, with MFI of PDCD4 mVenus being quantified using flow cytometry. As expected, we observed an MOI-dependent increase in PDCD4 degradation, with a more pronounced effect after 16 hours of infection (Fig. 3D). These results underscore the dynamic sensitivity of TOSI reporter cells, reaffirming their suitability for HTS aimed at detecting subtle alterations in mTOR activity.

### High-throughput screening for potential mTOR inhibitors using FDA-approved drugs

The mTOR signaling pathway is vital in various immune responses, including autophagy, positioning it as a critical target for host-directed therapies. The activation of mTOR during mycobacterial infections led us to investigate inhibitors of this pathway to deepen our understanding of mycobacterial influence on the host immune system. We employed TOSI reporter cells for high-throughput screening of an FDA-approved drug library, aiming to identify compounds that could inhibit mTOR activation in response to mycobacterial infection. To ensure the accuracy of our findings, we conducted duplicate screenings of 786 drugs in 96-well plates. Our library encompassed established mTOR inhibitors such as rapamycin, temsirolimus, and everolimus, serving as benchmarks to gauge the TOSI reporter assay’s sensitivity and specificity.

**Fig 4:**
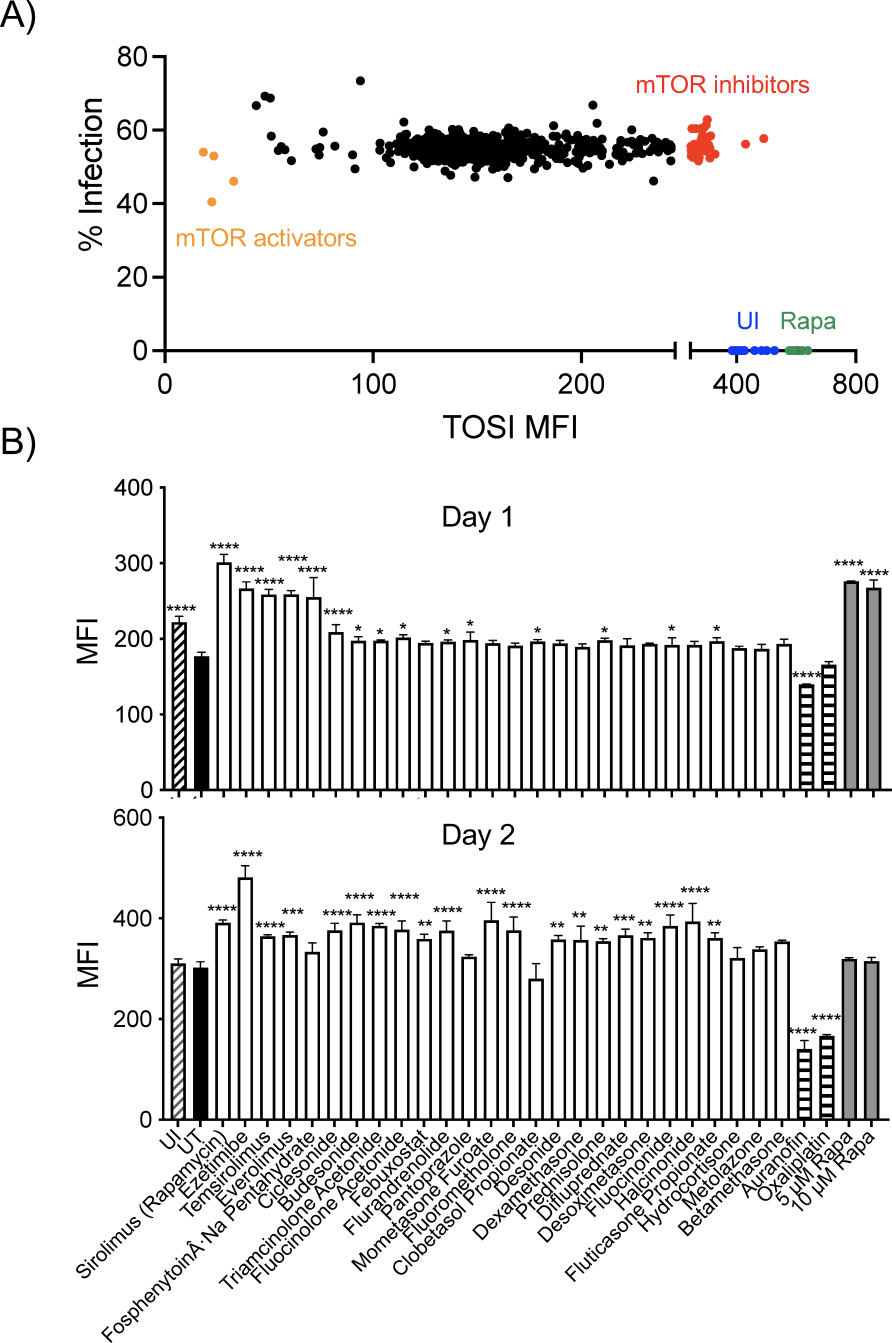
High-throughput screening of a drug library using TOSI cells. (**A**) A scatterplot from a representative experiment illustrates drugs that increased mVenus MFI, marked as red dots for further analysis. Green dots represent rapamycin-treated samples, blue dots represent uninfected cells, and orange dots represent mTOR-activating drugs. The consistency of infection across these samples was verified by evaluating tdTomato percentages in drug-treated cells. (**B**) The levels of PDCD4 were monitored in TOSI cells infected with *M. tuberculosis* H37Ra at an MOI of 10 and treated with selected drugs, indicated in gray. Auranofin and oxaliplatin, which exhibited low TOSI mVenus levels in preliminary screening, were used as negative controls and are represented in blue. Various concentrations of rapamycin, depicted in red, were employed as a positive control. These data represent the average of three replicates, expressed as the mean ± standard deviation (SD). Statistical significance was evaluated using a two-way ANOVA and Dunnett’s test for multiple comparisons. Significance thresholds were set at *P ≤ 0.05, **P ≤ 0.01, ***P ≤ 0.001, and ****P ≤ 0.0001.

In our experimental setup, we first infected TOSI cells with tdTomato-labeled *M. tuberculosis* H37Ra, followed by treatment with various compounds at a 10-µM concentration for 24 hours. PDCD4 levels in each well were quantified using the Opera system. Our control groups consisted of uninfected TOSI cells (blue dots) and uninfected cells treated with rapamycin (green dots), both demonstrating significant TOSI responses. As anticipated, compounds that effectively inhibited mTOR exhibited elevated PDCD4 levels, evident from the increased mVenus MFI compared to infected but untreated (UT) cells. The average mVenus MFI for UT cells was approximately 160 (Fig. 4A). Notably, well-known direct mTOR inhibitors like rapamycin, temsirolimus, and everolimus significantly increased PDCD4 levels. A significant inhibitory effect on mTOR was determined by an increase in mVenus MFI values exceeding twice the standard deviation above the mean for the entire drug library (red dots), indicative of PDCD4 stabilization.

From the primary screening, we identified the top 50 drugs with the highest mVenus MFI values relative to the UT group, further narrowing down to 26 drugs for a second round of screening (Table 1). This shortlist featured 18 corticosteroids, commonly used to treat various inflammatory conditions and allergies. To validate these preliminary results, we employed Opera imaging and Western blot analyses in the secondary screening, which reconfirmed enhanced mTOR inhibition with the selected drugs. Compounds such as everolimus and temsirolimus consistently inhibited mTOR. Other drugs also exhibited increased inhibition relative to UT samples, particularly after 2 days of treatment (Fig. 4B). We included negative controls, auranofin and oxaliplatin, which displayed low mVenus levels (orange dots), to validate the screening process. This observation suggests potential indirect mTOR inhibition, resulting in a delayed response compared to direct inhibitors. Our findings highlight the effectiveness of TOSI cells as reporter lines for high-throughput screening, pinpointing their significant role in identifying mTOR inhibitors or activators and their promising application in drug discovery.

**TABLE 1:** Selected drugs for second-round screening^*a*^

Name of drug	Classification
**Ezetimibe**	Cholesterol absorption inhibitor and a dyslipidemic agent
**Sirolimus (rapamycin**)	mTOR inhibitor belonging to macrolide lactams
**Temsirolimus**	Derivative of Sirolimus, mTOR inhibitor belonging to macrolide lactams
**Everolimus**	Derivative of Sirolimus, mTOR inhibitor belonging to macrolide lactams
**Budesonide**	Corticosteroid
**Desonide**	Topical synthetic corticosteroid
**Pantoprazole**	Belongs to the benzimidazole group of proton pump inhibitors
**Triamcinolone acetonide**	Glucocorticoid
**Ciclesonide**	Nonhalogenated corticosteroid
Metolazone	Quinazoline sulfonamide diuretic that belongs to the thiazide class
Fluocinolone acetonide	Topical corticosteroid
Clobetasol propionate	Corticosteroid
Prednisolone	Synthetic, anti-inflammatory glucocorticoid derived from cortisone
Febuxostat	Non-purine selective inhibitor of xanthine oxidase inhibitor
Fluorometholone	An ophthalmic corticosteroid
Halcinonide	A topical corticosteroid
Fosphenytoin·Na pentahydrate	An ester prodrug of phenytoin
Flurandrenolide	Topical corticosteroid
Dexamethasone	Glucocorticoid
Fluticasone propionate	Glucocorticoid
Difluprednate	Topical anti-inflammatory corticosteroid
Mometasone furoate	Synthetic steroid hormone of glucocorticoid family
Betamethasone	Corticosteroid
Desoximetasone	Glucocorticoid
Fluocinonide	Topical corticosteroid
Hydrocortisone	Synthetic steroid belonging to glucocorticoid family

^*a*^ Drugs highlighted in bold are used for Western blotting.

### Selected drug candidates demonstrate suppression of phosphorylated S6

The mTOR pathway plays a pivotal role in cellular growth, proliferation, and survival by sensing and integrating various environmental cues. A key effector of this pathway is S6 Kinase 1 (S6K1), which is directly phosphorylated by mTOR. Once activated, S6K1 phosphorylates the S6 ribosomal protein, leading to its phosphorylated form (p-S6). This phosphorylation event is not just a downstream consequence of mTOR activation but also critically involved in the translation of specific mRNA subsets that encode components essential for cell growth and division. Thus, p-S6 serves as a reliable biomarker for mTOR activity, reflecting the functional state of a central growth-regulating pathway (37).

**Fig 5:**
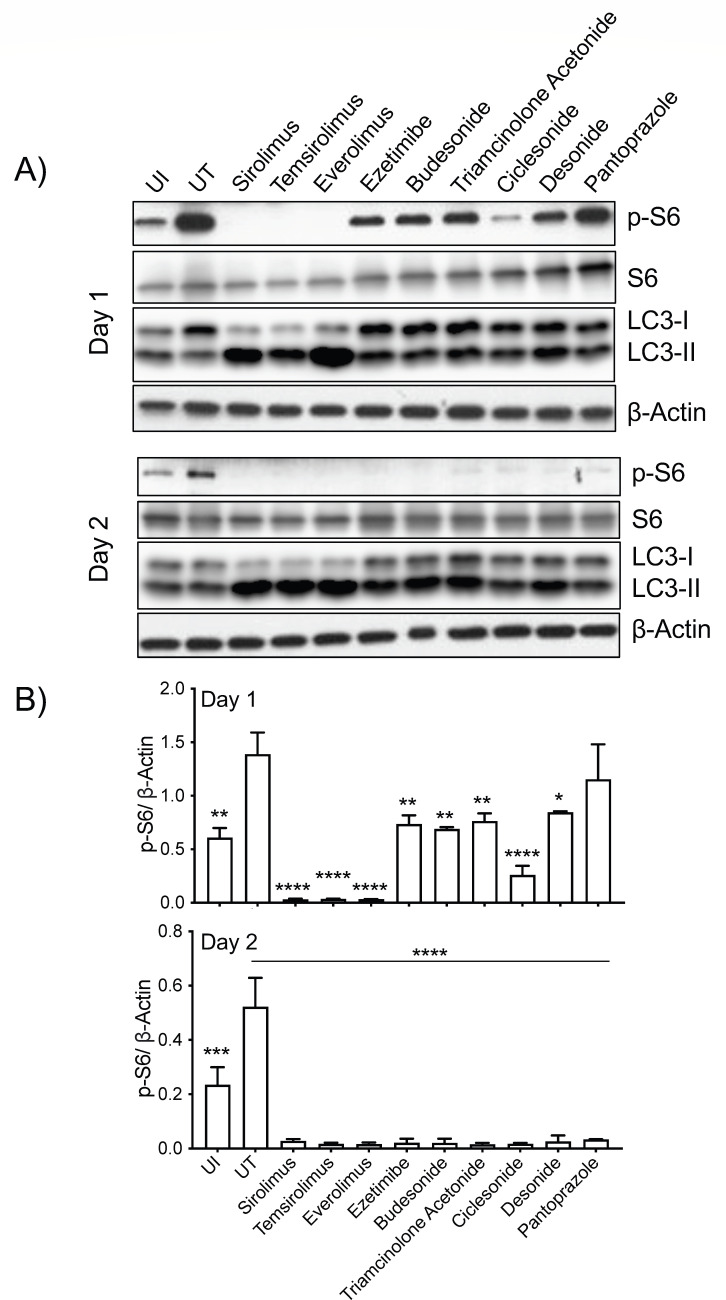
Immunoblot analysis of TOSI cell lysates post-drug treatment and mycobacterial infection. (**A**) TOSI cells, treated with a range of drugs and infected with *M. tuberculosis* H37Ra, underwent immunoblot analyses 1 and 2 days post-infection. These analyses revealed the levels of pS6 and LC3, utilizing β-actin as a loading control. (**B**) Densitometric analysis of the data acquired from Figure 5A was performed, with normalization to β-actin to evaluate phosphorylated S6 (P-S6). Statistical significance was assessed using a two-way ANOVA and Dunnett’s test for multiple comparisons. Significance levels were indicated as *P ≤ 0.05, **P ≤ 0.01, and ****P ≤ 0.0001.

To confirm our screening results, we conducted a Western blot analysis on infected TOSI cells, evaluating pS6 levels post-treatment with selected drugs. We chose a range of drug candidates including sirolimus (rapamycin), temsirolimus, everolimus, ezetimibe, budesonide, triamcinolone acetonide, ciclesonide, desonide, and pantoprazole (Fig. 5A). Our results showed a marked decrease in phosphorylated S6 at days 1 and 2 compared to the UT control. This suggests effective inhibition of the mTOR pathway by these drugs (Fig. 5B). Concurrently, we observed an increase in autophagic response in infected cells treated with mTOR inhibitors, as evidenced by elevated expression of LC3B-II starting just 1 day after initiating treatment and increasing further by day 2 (Fig. S7). These findings underscore the potential of these drugs as autophagy activators and highlight their promise in enhancing the clearance of mycobacterial infections through host-directed therapeutic strategies. This comprehensive approach, combining the analysis of pS6 as a direct marker of mTOR pathway modulation with autophagy markers, provides a robust framework for evaluating the efficacy of these drugs in a cellular context.

### Inhibition of mTOR significantly restricts mycobacterial growth within macrophages

**Fig 6:**
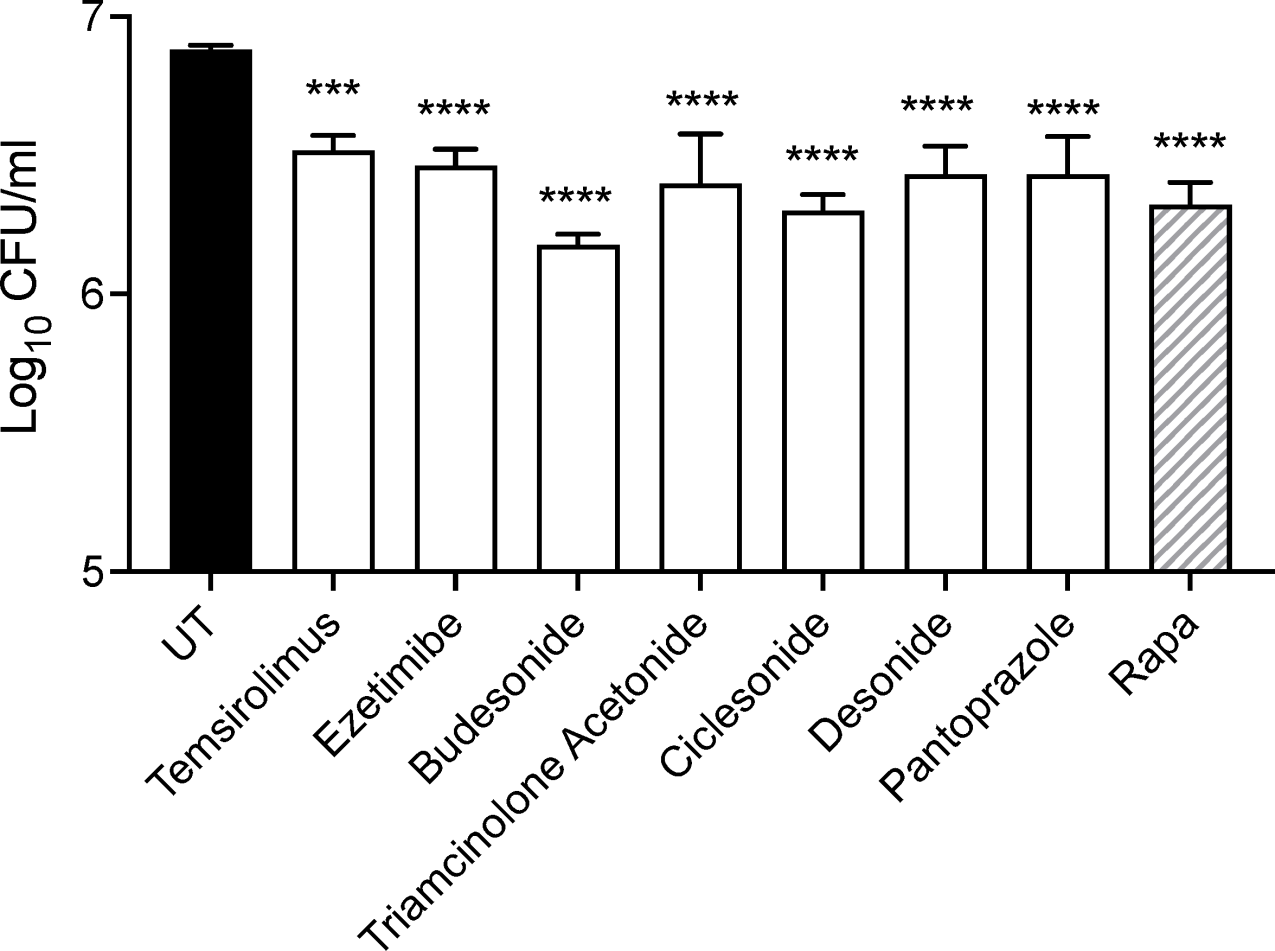
Inhibition of mycobacterial growth in macrophages by mTOR inhibitors. The intracellular growth of *M. tuberculosis* H37Ra in TOSI cells was evaluated after treatment with mTOR inhibitors. The cells were infected at an MOI of 10 and then treated with drugs at a concentration of 10 µM. Bacterial growth was quantified using CFU measurements 2 days post-treatment. The data, presented as mean ± SD, are derived from one of two independent experiments. The statistical significance of the results was established through a two-way ANOVA, augmented by Dunnett’s test for conducting multiple comparisons. Notably, levels of significance were marked as ***P ≤ 0.001 and ****P ≤ 0.0001.

Targeting mTOR inhibition was explored as a strategy to enhance bacterial clearance in host-directed therapeutic applications. We treated TOSI cells, infected with *M. tuberculosis* H37Ra at an MOI of 10, with mTOR inhibitors identified from our screening. This treatment led to a notable increase in LC3-II expression, indicative of an augmented autophagy-mediated host-directed therapeutic response. Remarkably, a substantial decrease in bacterial load was observed in treated cells compared to infected but untreated cells after 2 days of treatment (Fig. 6).

Our results align with prior studies indicating that corticosteroids provide cytoprotective effects in *M. tuberculosis*-infected cells and diminish bacterial loads in human monocyte-derived macrophages (38, 39). Previous research has shown that ezetimibe can effectively reduce the intracellular load of mycobacteria. Additionally, the use of ezetimibe in diabetic patients has been associated with a decreased risk of developing latent tuberculosis infection (40). Similarly, pantoprazole, a proton pump inhibitor, has been identified for its capacity to inhibit macrophage-induced rifampicin tolerance and intramacrophage growth of *M. tuberculosis* (41). These findings validate the efficacy of the selected mTOR inhibitors in limiting mycobacterial growth in macrophages, underscoring their significant potential for future research into treatment strategies for mycobacterial infections.

## DISCUSSION

PDCD4, a protein primarily recognized for its central roles in cancer and immune regulation, has garnered significant attention as a therapeutic target. Its applications are diverse, extending from cancer treatment to managing metabolic conditions like polycystic ovary syndrome, obesity, and diabetes (42–46). As an important cell cycle regulator, PDCD4 is involved in the regulation of various cell processes such as apoptosis; its expression is upregulated during apoptosis, while downregulation of PDCD4 also activates apoptotic pathways (33, 34, 47–50). Notably, monocytes infected with *Mycobacterium leprae* show an upregulation of miRNA-21, a negative regulator of PDCD4 (51). However, the dynamics of PDCD4 in other mycobacterial infections remain to be explored. Our research reveals that mycobacterial activation of the mTOR pathway triggers PDCD4’s downregulation. Our findings indicate a downregulation of PDCD4 during mycobacterial infection, urging further exploration into the underlying factors. This indicates its suitability as an alternative marker for gauging mTOR activation during mycobacterial infections, providing a new perspective in understanding these complex biological interactions.

Traditional methods such as Western blotting, although useful, have limitations in scalability and quantitative analysis. These constraints make them less suited for developing therapies targeting mTOR inhibition. To address these limitations, we leveraged PDCD4 as a biomarker for mTOR inhibition. In this context, we employed TOSI reporter cell lines, which have demonstrated greater sensitivity compared to traditional methods. This approach was crucial in identifying potential mTOR inhibitors and efficiently screening various compounds (Fig. 4A).

In our study, we identified mTOR inhibitors as promising agents for the treatment of mycobacterial infections. These inhibitors, especially those in the rapamycin class, demonstrated dual effectiveness by enhancing PDCD4 expression and simultaneously reducing bacterial load in macrophages. A notable observation was the potent induction of autophagy by rapamycin derivatives, likely attributable to their direct inhibitory action on mTOR. This was evidenced by the heightened autophagic response, characterized by increased LC3-II expression. Other novel compounds evaluated, such as triamcinolone acetonide and ciclesonide, also showed similar autophagic activity in *M. tuberculosis*-infected macrophages, suggesting their potential role as anti-mycobacterial agents. However, the interpretation of these findings requires caution. The mTOR pathway is known for its intricate network of interactions influencing various cellular processes. Inhibition of mTOR can therefore lead to diverse, sometimes unpredictable cellular outcomes. This complexity necessitates a comprehensive evaluation of the wider physiological impacts of these mTOR inhibitors. Although these initial findings are encouraging, they highlight the importance of continued research to fully understand the implications and therapeutic potential of these drugs in the context of mycobacterial infection treatment.

In our study, as illustrated in Figure 5 and 6, a range of corticosteroids were identified as potential inhibitors of the mTOR pathway. These corticosteroids, often used in conjunction with TB treatments, have been recognized for their role in reducing bacterial growth and minimizing tissue damage. They achieve this by inducing cytoprotective responses. The way corticosteroids modulate the immune system and influence the mTOR pathways suggests a significant potential impact on various immune processes (52). The underlying mechanism by which corticosteroids may affect mTOR signaling is hypothesized to involve their interaction with specific receptors, which in turn could alter components of the mTOR pathway (53). Such interactions are believed to result in either the enhancement or suppression of mTOR pathway activity, varying according to the specific cell type and the contextual environment.

Furthermore, glucocorticoids, a type of corticosteroid that notably influences glucose metabolism, have significant anti-inflammatory and immunosuppressive effects. These substances have been shown to suppress mTOR signaling in diverse cell types, including lymphoid cells, skeletal muscle, hypothalamic organotypic cultures, and primary cortical neurons. Interestingly, prior stress exposure seems to affect mTOR balance, complicating the maintenance of equilibrium when glucocorticoids are introduced later. This can skew mTOR signaling toward enhanced cell death pathways (54, 55). Conversely, there is evidence of glucocorticoids activating mTORC1 in certain cell types, playing a critical role in regulating functions such as protein synthesis, metabolism, and cell survival. For instance, dexamethasone is known to inhibit autophagy, possibly via the inhibition of the mTORC1 pathway (56). This differential response might be attributed to the variation in stress sequence and drug dosage. For example, in one study, macrophages were pre-treated with 1 µM dexamethasone before BCG infection, contrasting our approach where macrophages infected with *M. tuberculosis* H37Ra were subsequently treated with 10 µM drugs. The impact of glucocorticoids on mTOR and autophagy seems to be complex and cell-type dependent. For example, glucocorticoids were found to enhance osteoclast autophagy through the PI3K/Akt/mTOR signaling pathway (57). Another study highlighted their variable effects on the hippocampus, influenced by factors like stress history (58).

Our research highlights the critical roles of both mTOR signaling and corticosteroids in metabolic regulation, emphasizing their complex interplay across various tissues and disease states. We have uncovered a potential role for PDCD4 in mediating the therapeutic effects of corticosteroids, suggesting an indirect mechanism for mTOR inhibition by these agents. This finding opens exciting prospects for their synergistic use with existing anti-TB treatments. Furthermore, our study spotlights the significance of PDCD4 in mycobacterial infections and its potential as a marker of mTOR activity. The effectiveness of the TOSI reporter in drug screening underscores the importance of inhibitors that restrict mycobacterial growth, thereby underlining the growing relevance of PDCD4 in bacterial infections and indicating promising avenues for future therapeutic developments.

## SUPPLEMENTAL MATERIAL

Supplemental figures spectrum.00062-24-s0001.pdf Fig. S1–S7.

ASM does not own the copyrights to Supplemental Material that may be linked to, or accessed through, an article. The authors have granted ASM a non-exclusive, world-wide license to publish the Supplemental Material files. Please contact the corresponding author directly for reuse.
