# Supplementary material for: PDCD4 as a marker of mTOR pathway activation and therapeutic target in mycobacterial infections
Source: Microbiol Spectr. 2024 Jun 24;12(8):e00062-24. doi: 10.1128/spectrum.00062-24 (PMC11302300; doi:10.1128/spectrum.00062-24)
Supplement: Supplemental figures — Fig. S1–S7. [file spectrum.00062-24-s0001.pdf]

Fig. S1. Schematic Depiction of Mycobacterial-Induced mTOR Activation.

This figure provides a detailed schematic representation of the molecular pathway activated by mycobacterial infection, emphasizing the activation of the mammalian target of rapamycin (mTOR) signaling pathway. The mTORC1 complex is activated in response to various stimuli, including growth factors, nutrients, and cellular energy levels. This activation sets off a cascade of signaling pathways that regulate protein synthesis, as well as cell growth and survival. When mTORC1 is activated, it leads to the phosphorylation and activation of S6K1. Activated S6K1 then phosphorylates ribosomal protein S6 and other targets, boosting the translation of specific mRNAs and thereby enhancing protein synthesis. PDCD4, a tumor suppressor that impedes translation, is phosphorylated by the activated S6K1. This phosphorylation, facilitated by S6K1 under the influence of mTORC1, occurs at designated sites on PDCD4. Once PDCD4 is phosphorylated, it undergoes ubiquitination, a process in which ubiquitin is attached to a protein, signaling it for degradation. This phosphorylated PDCD4 is then identified and degraded by the cell's proteasome. The degradation of PDCD4 reduces its inhibitory effect on protein synthesis. As a result, the presence and levels of PDCD4 serve as an inverse indicator of mTORC1 activity.

Fig. S2. Live dead staining of TOSI cells. TOSI cells were either uninfected/untreated (UI/UT), infected with *M. smegmatis* (Msm) at an MOI of 5 for 3 hours, or treated with 1 μg/ml rapamycin overnight. The cells were stained using FVD660 to assess viability and analyzed using a BD Symphony flow cytometer. The accompanying graph illustrates the gating strategy for each group. Finally, histograms of the TOSI signal were overlaid to compare the responses.

Fig. S3. Time-Dependent Reduction in PDCD4 Expression. TOSI cells infected with *M. smegmatis* (Msm) at a multiplicity of infection (MOI) of 10 showed a time-dependent reduction in PDCD4 expression. The cells were lysed at various time points post-infection and immunoblots were conducted to analyze the expression of PDCD4, pS6, and LC3-II in the cell lysates. β-actin was employed as a loading control.

Fig. S4. Differential Expression of PDCD4 During Mycobacterial Infection. Differential expression of PDCD4 in TOSI cells, infected with *M. smegmatis* (Msm) and *M. tuberculosis* H37Ra (Mtb) at a multiplicity of infection (MOI) of 10, is demonstrated. The cells were fixed at time intervals of 0, 3, 8, 24, 36, and 48 hours post-infection, and mVenus MFI was measured using the Opera high-content imaging system. Rapamycin was used as a positive control in this analysis.

Fig. S5. Densitometric Measurement of PDCD4, pS6 and LC3-II upon mycobacterial infection. Expression levels of different proteins, including PDCD4, pS6, and LC3-II, were quantified from the blots shown in Figure 2C using ImageJ. The statistical significance of the results was determined through One-way ANOVA, supplemented by Dunnett's Test for conducting multiple comparisons. Levels of significance were denoted as $*p \leq 0.05$, $**p \leq 0.01$, $***p \leq 0.001$, and $****p \leq 0.0001$.

Fig. S6. Z'-Factor Calculation for High-Throughput Screening Validation. To validate the high-throughput screening process, we employed 20 replicates for each experimental condition: one set of uninfected TOSI cells and another treated with 5 μM rapamycin for a duration of up to

3 hours. We measured the mVenus MFI using the Opera high-content imaging system. For the calculation of the Z'-factor, we used these mVenus MFI values, following the formula outlined in the materials and methods section of the study.

Fig. S7. Analysis of LC3-II in TOSI Cell Lysates Following Drug Treatment and Mycobacterial Infection. Densitometric analysis was performed on the data from Figure 5A, with normalization to β-actin to assess the levels of LC3-II. Statistical significance was assessed using a two-way ANOVA and Dunnett's Test for multiple comparisons. Significance levels were indicated as *$p \leq 0.05$, **$p \leq 0.01$, ***$p \leq 0.001$, and ****$p \leq 0.0001$.

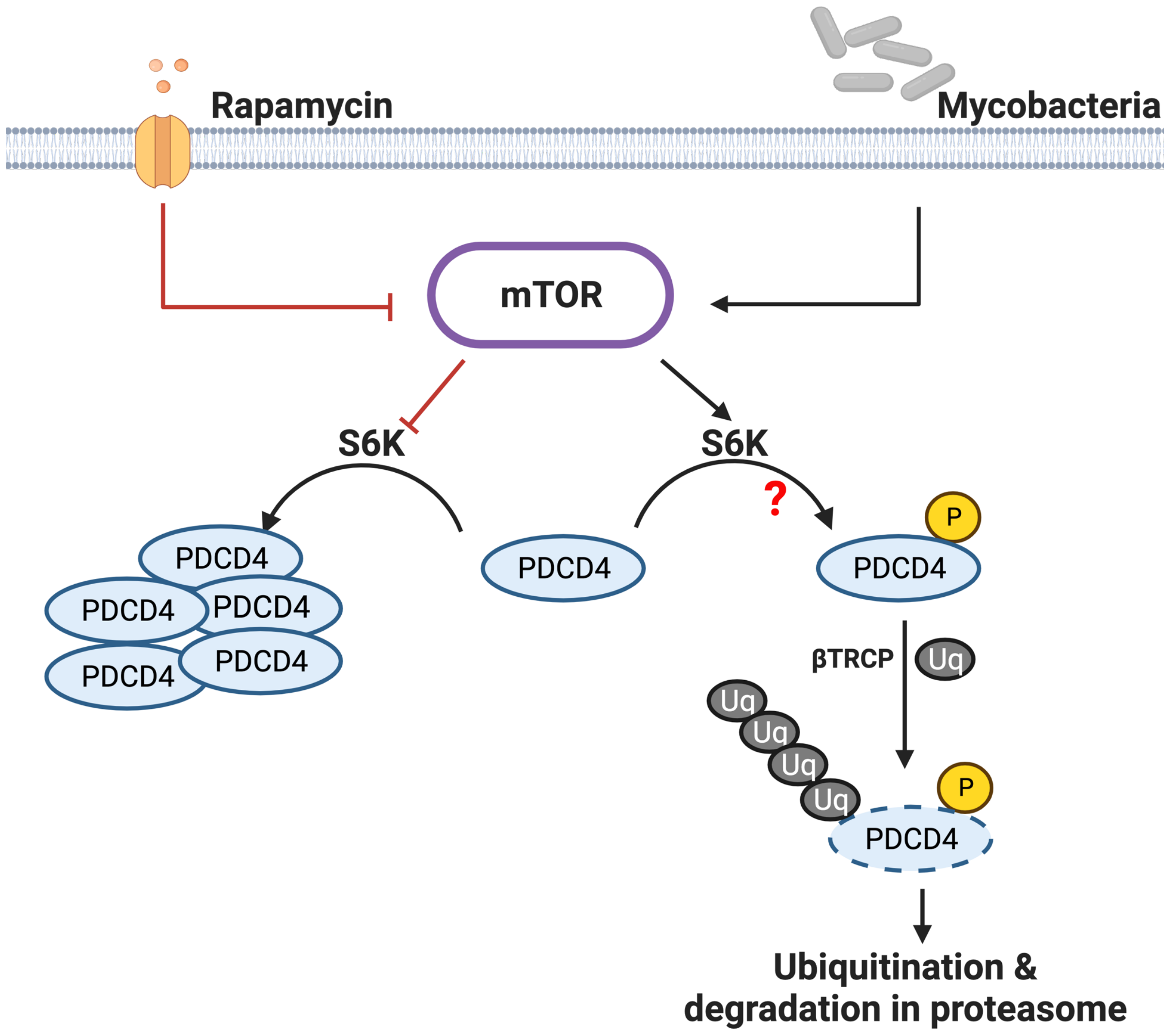
Rapamycin
Mycobacteria
mTOR
S6K
S6K
?
PDCD4
PDCD4
PDCD4
PDCD4
PDCD4
PDCD4
P
PDCD4
βTRCP
Uq
Uq
Uq
Uq
Uq
P
PDCD4
Ubiquitination &
degradation in proteasome

Fig. S1

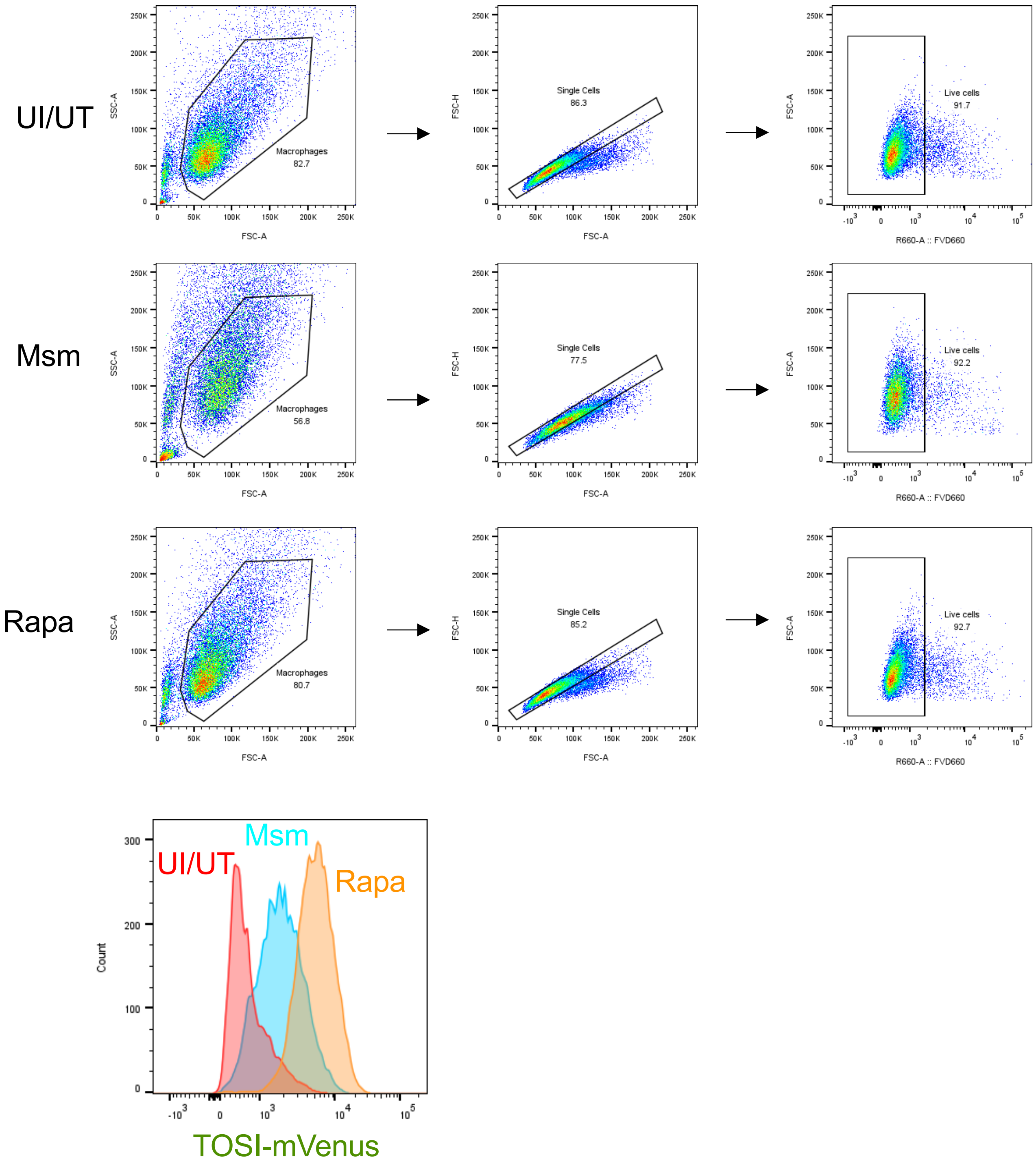
UI/UT
Msm
Rapa
SSC-A
FSC-A
FSC-H
R660-A :: FVD660
Macrophages
82.7
56.8
80.7
Single Cells
86.3
77.5
85.2
Live cells
91.7
92.2
92.7
Count
TOSI-mVenus

Fig. S2

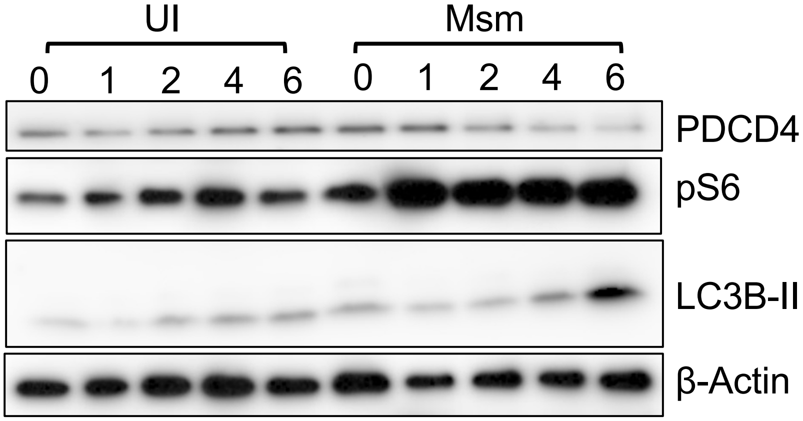

Fig. S3

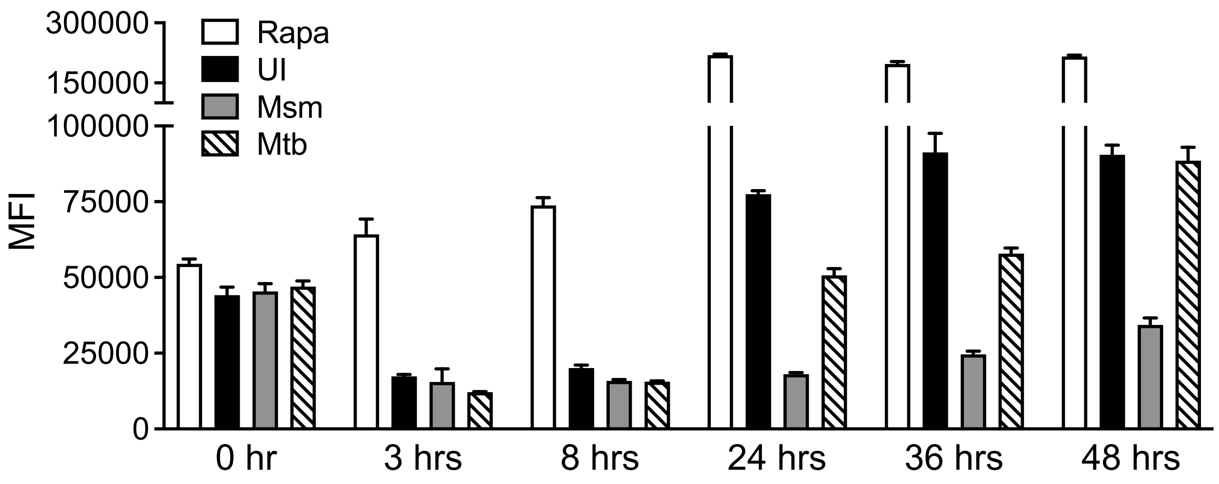
300000
150000
100000
75000
50000
25000
0
MFI
Rapa
UI
Msm
Mtb
0 hr
3 hrs
8 hrs
24 hrs
36 hrs
48 hrs

Fig. S4

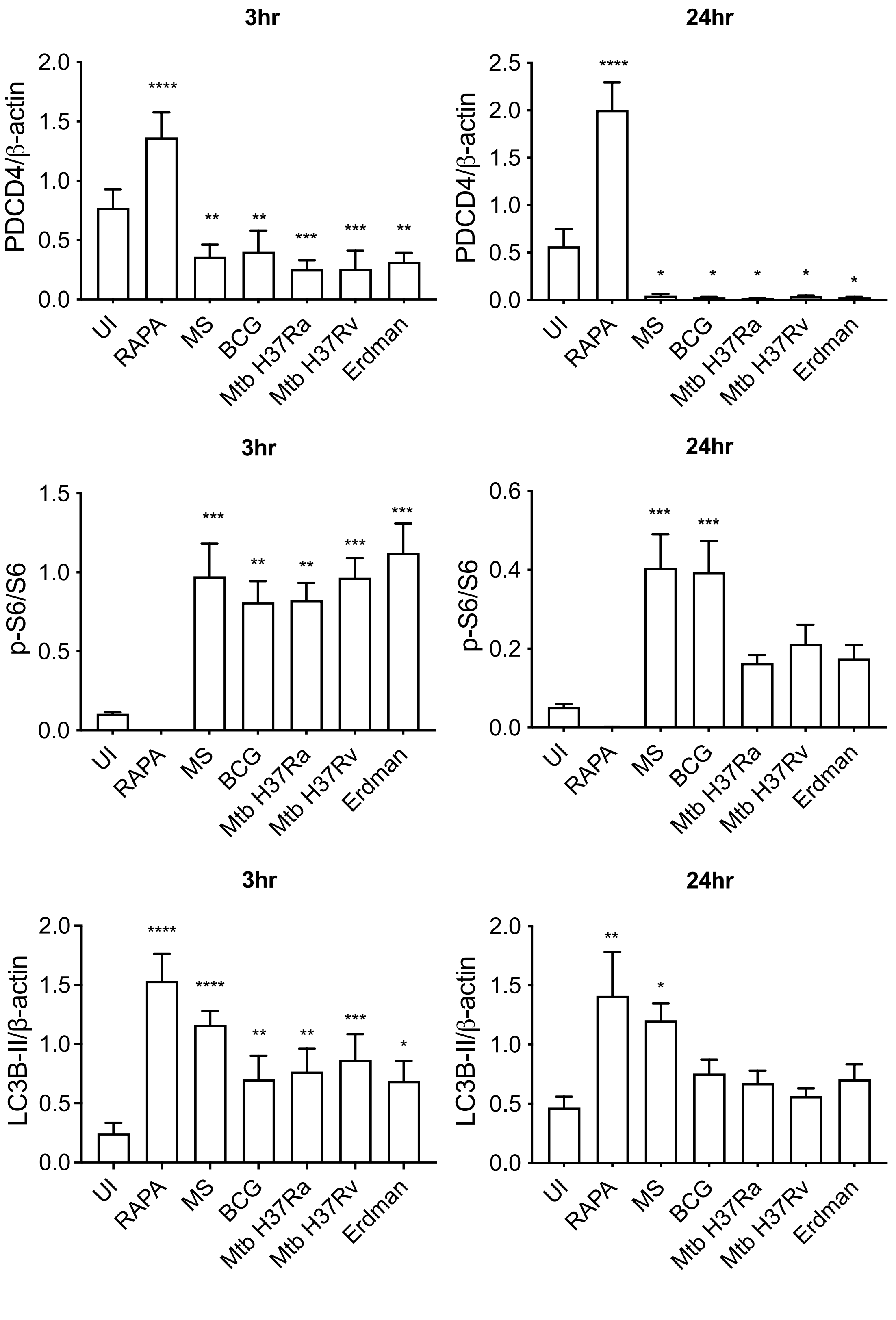
3hr
24hr
PDCD4/β-actin
p-S6/S6
LC3B-II/β-actin
UI
RAPA
MS
BCG
Mtb H37Ra
Mtb H37Rv
Erdman
****
***
**
*

Fig. S5

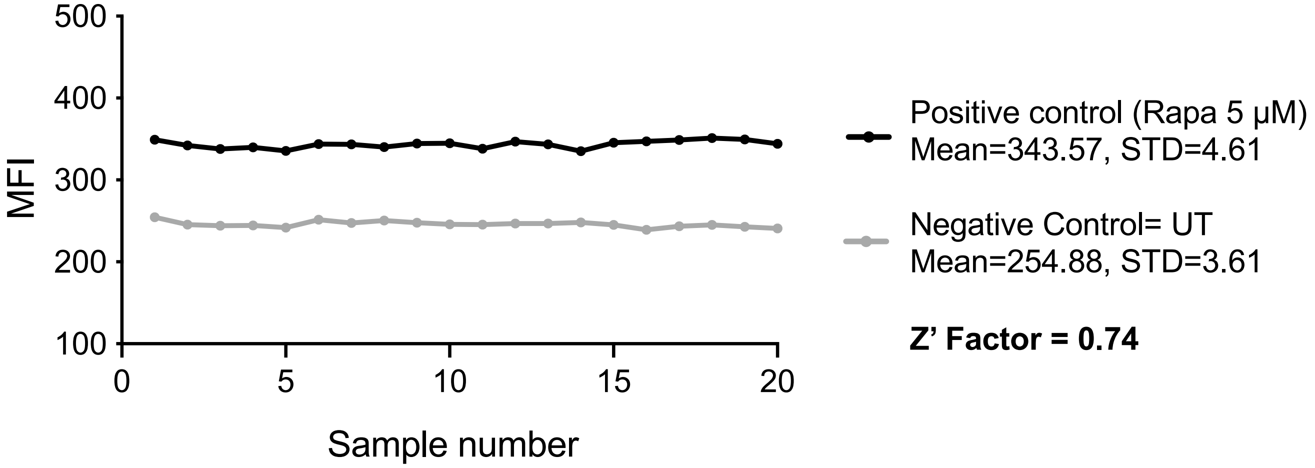
500
400
300
200
100
MFI
0
5
10
15
20
Sample number
Positive control (Rapa 5 μM)
Mean=343.57, STD=4.61
Negative Control= UT
Mean=254.88, STD=3.61
Z' Factor = 0.74

Fig. S6

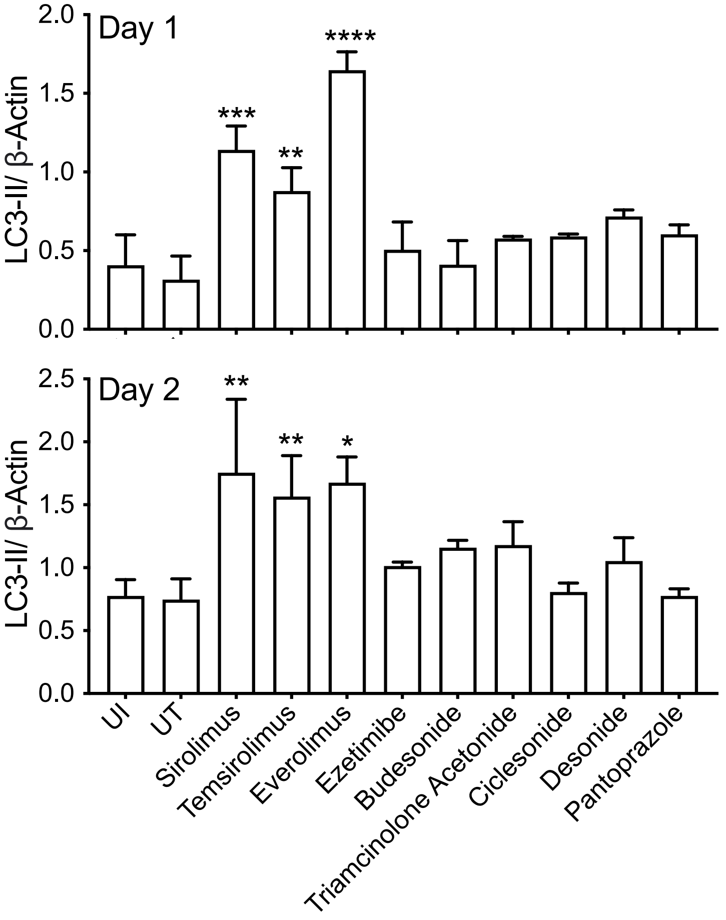
Day 1
LC3-II/ β-Actin
2.0
1.5
1.0
0.5
0.0
***
**
****
Day 2
LC3-II/ β-Actin
2.5
2.0
1.5
1.0
0.5
0.0
**
**
*
UI
UT
Sirolimus
Temsirolimus
Everolimus
Ezetimibe
Budesonide
Triamcinolone Acetonide
Ciclesonide
Desonide
Pantoprazole

Fig. S7
